# From Reductionism Toward Integration: Understanding How Social Behavior Emerges From Integrated Circuits

**DOI:** 10.3389/fnint.2022.862437

**Published:** 2022-04-01

**Authors:** Sarah Y. Dickinson, Diane A. Kelly, Stephanie L. Padilla, Joseph F. Bergan

**Affiliations:** ^1^Neuroscience and Behavior Program, University of Massachusetts Amherst, Amherst, MA, United States; ^2^Department of Psychological and Brain Sciences, University of Massachusetts Amherst, Amherst, MA, United States; ^3^Department of Biology, University of Massachusetts Amherst, Amherst, MA, United States

**Keywords:** social behavior, circuit, neuromodulation, sensory input, behavior, integration

## Abstract

Complex social behaviors are emergent properties of the brain’s interconnected and overlapping neural networks. Questions aimed at understanding how brain circuits produce specific and appropriate behaviors have changed over the past half century, shifting from studies of gross anatomical and behavioral associations, to manipulating and monitoring precisely targeted cell types. This technical progression has enabled increasingly deep insights into the regulation of perception and behavior with remarkable precision. The capacity of reductionist approaches to identify the function of isolated circuits is undeniable but many behaviors require rapid integration of diverse inputs. This review examines progress toward understanding integrative social circuits and focuses on specific nodes of the social behavior network including the medial amygdala, ventromedial hypothalamus (VMH) and medial preoptic area of the hypothalamus (MPOA) as examples of broad integration between multiple interwoven brain circuits. Our understanding of mechanisms for producing social behavior has deepened in conjunction with advances in technologies for visualizing and manipulating specific neurons and, here, we consider emerging strategies to address brain circuit function in the context of integrative anatomy.

## Introduction

Technical progress in neuroscience has accelerated the capacity for resolving complex circuits with high resolution. Many of the first circuits studied in terms of both connectivity and function were small, consisting of a few dozen cells, often from invertebrate species (Selverston et al., 1976; Russell, 1979; Katz et al., 1989; Marder and Bucher, 2001). Neuroscientists studying larger circuits in vertebrates adapted the logic and conceptualizations advanced by invertebrate researchers to larger circuits (Katz et al., 2013); however, each node in vertebrate circuit diagrams typically represented a brain region containing thousands or millions of neurons. This translation was immediately effective for brain regions with repeating motifs (e.g., the retina or primary visual cortex), however, other more heterogeneous brain regions encompassed multiple overlapping and interleaved circuits, each with different functions. For example, the ventromedial nucleus of the hypothalamus emerged as a focal point for thermogenesis as well as hunger and aggression (Perkins et al., 1981; Lin et al., 2011; Gaur et al., 2014). Similarly, the medial amygdala (MeA) was linked to reproduction, parenting, aggression, and same-sex affiliative behaviors (Lehman et al., 1980; Unger et al., 2015; McCarthy et al., 2017; Chen et al., 2019; Wu et al., 2021). Here, we discuss the role of technological innovation in identifying and isolating MeA circuits specific for precise behaviors and then discuss the growing appreciation for, and need to understand, the role of broadscale integration as an organizing principle of social circuit design.

Social behaviors are critically important for survival in species throughout all branches of the animal kingdom. In vertebrates, the social behavior network (SBN) is a conserved network of brain regions essential for social behaviors. Foundational work established links between specific brain regions in the SBN and behaviors such as aggression, affiliation, reproduction, and parenting (Rosvold et al., 1954; Lammers et al., 1988; Newman, 1999). The picture emerging from these studies and subsequent research was one of dense connectivity between nodes of the SBN, with each social behavior controlled by multiple brain regions and each node of the SBN contributing to the regulation of multiple social behaviors. The interwoven nature of social behaviors and the circuits that mediate them represented an immediate challenge for neuroscientists interested in understanding the neural control for a specific social behavior. For example, electrolytic lesions lack specificity for genetic cell-type, and consequently indiscriminately impacted all neurons at the lesion site. Similarly, with few exceptions (Margolis et al., 2006) electrophysiological recordings lacked specificity for genetically defined cell-types and, accordingly, were often poorly equipped to distinguish between anatomically overlapping circuits (Chen and Toney, 2010; Bergan et al., 2014).

**FIGURE 1:**
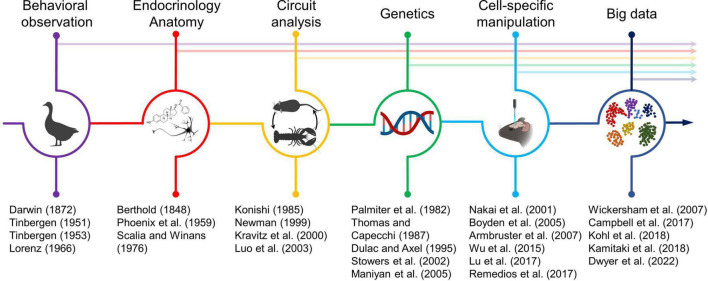
Timeline of the dynamic relationship between technological innovation and biological insight in neuroscience. Technical trends that have profoundly shaped mechanistic insight for social neuroscience are highlighted at the top. Arrows indicate that most developed technologies continue to shape neuroscience to this day. Key papers describing either the development of a technical innovation or the adaptation of a new technique for investigating brain function are listed at the bottom. Select icons adapted from BioRender.com.

Social neuroscience, as a formal field of study, dates at least as far back as the work of early neuroethologists that sought to study how the brain interprets sensory cues and guides natural behaviors in a wide array of species. Progress toward understanding the neural circuit principles that mediate behavior has required adapting a wide array of concepts and techniques including careful behavioral observation, endocrinology, anatomy, electrophysiology, genetics, optogenetics and chemogenetics, single cell sequencing, and connectomics along with many other strategies (Figure 1). During the latter half of the twentieth century technological advances in monitoring neurons, genetically targeting cells, manipulating neurons, investigating neural circuits, and extracting insight from large datasets collectively ushered in a new era for identifying and causally testing the precise circuits necessary for specific social behaviors.

The capacity for targeted lesions has, in some cases, reached the level of single cells (e.g., Ruta et al., 2010) and pharmacological agents increasingly allow specific targeting of single channels (e.g., Alexander et al., 2010). Advances in single cell sequencing are revealing the heterogeneity of neuron populations and are allowing neuroscientists to define a “parts list” required for circuit function that accounts for structural and molecular heterogeneity in the cells that constitute each circuit (Macosko et al., 2015; Hrvatin et al., 2017; Kamitaki et al., 2018). The introduction of optogenetics and chemogenetics make it possible to activate and inhibit circuits reversibly with high temporal precision (Boyden et al., 2005; Alexander et al., 2010). This, in combination with advances in *in vivo* electrophysiology (Lima et al., 2009; Cohen et al., 2012), and advances in optical imaging of neural activity (Kim et al., 2016; Li et al., 2017) allows neuroscientists to probe questions with novel behavioral resolution and strengthen links between behaviors and the circuits that mediate them. As the precision of tools improved, the resolution with which individual behaviors were understood increased proportionally (Figure 1). However, the interwoven nature of social circuits is likely an important design principle that allows integration of diverse sensory and interoceptive inputs to produce behavior well-adapted to the individual’s environment. We suggest that the same tools that accelerated insight into cell-type specific neural circuits specialized for specific social behaviors are also essential for understanding how emergent properties of social behavior are mediated by the connections between circuits and the broad integration of sensory inputs and internal states.

## Isolating Neural Circuits Underlying Behavior

Untangling the intermingled circuits in the SBN depends on identifying each reliable and reproducible subset of neurons whose activity produces a specific behavior. For example, territorial aggression is triggered by sensory cues unique to each species (Tinbergen, 1951; Harrison, 1983; Goodson and Kabelik, 2009; Butler and Maruska, 2015), with chemosensory stimuli being particularly salient in rodents (Schultz and Tapp, 1973; Stowers et al., 2002; Hubbard et al., 2004; Mandiyan et al., 2005). Aggression-inducing cues are detected by both the main olfactory and vomeronasal system and conveyed to a conserved circuit for aggression that includes the ventromedial hypothalamus (VMH) as a core component. Electrical stimulation of VMH directly produces aggression (Kruk et al., 1983); however, the VMH is also critical for satiety as lesions to the VMH produce over-eating and obesity as well as sexual behaviors (Musatov et al., 2006, 2007; Xu et al., 2011; Correa et al., 2015; Yang et al., 2017; Kammel and Correa, 2020). Disentangling these interleaved behavioral circuits requires access to genetically defined cell-type specific tools to enable reproducible targeting of precise VMH subcircuits. The advent of optogenetics (Zemelman et al., 2002; Boyden et al., 2005) further established the causal relationship of the ventrolateral VMH (VMHvl) as a control center for aggression. Specific activation of VMHvl directly produced aggression regardless of the social partner (even toward an inanimate object) while silencing VMHvl suppressed the level of territorial aggression toward intruders (Lin et al., 2011). Later, VMHvl neurons expressing either estrogen receptor alpha or progesterone receptor, likely overlapping populations, were identified as central populations required for VMHvl-induced aggression (Yang et al., 2017; Hashikawa et al., 2018). Importantly, the moment-to-moment neural activity of steroid responsive subpopulations of VMHvl neurons correlates exquisitely with aggressive encounters (Falkner et al., 2014). Collectively, these studies demonstrate a clear progression through which technical innovation, specifically the advent of cell type-specific manipulations identified causal relationships between precise populations of hypothalamic neurons and aggression.

Like advances in our understanding of aggression, technical advances have enabled rapid progress in understanding the neural basis of parental behavior. The medial preoptic area of the hypothalamus (MPOA) is linked to parental behavior, sexual behavior, and sleep. As above, these interwoven circuits were disentangled through the identification of a genetic marker, galanin, that is expressed in MPOA neurons specific for parental behavior. Activation of galanin-expressing neurons promotes parental behavior, while ablating galanin-expressing neurons suppresses it and, consequently, promotes infanticidal behaviors (Wu et al., 2014). Remarkably, phenotypic heterogeneity of subpopulations of galanin-expressing neurons maps on to specific behavioral features that collectively constitute parental behaviors—for example, one sub-population of galanin-expressing neurons suppresses interactions with other adults and a separate subpopulation of galanin-expressing neurons enhances motivation to interact with offspring (Kohl et al., 2018). Like the circuits required for aggressive behaviors, molecular and genetic tools were essential to isolate the circuits controlling parental behavior in the MPOA. While the behavioral output of these circuits is clear, the computations required to reach a behavioral “decision” integrate interoceptive, sensory, neuromodulatory, and developmental inputs.

## Circuits Mediating Behavioral Complexity in the Medial Amygdala

Both the VMHvl and MPOA nodes receive direct input from the MeA (Dong and Swanson, 2004; Kohl et al., 2018) that itself contains interwoven circuits devoted to nearly all social behaviors. The MeA, MPOA, and VMH are each core members of the SBN with the MeA being the primary recipient of chemosensory input, arriving directly from the accessory olfactory bulb (Scalia and Winans, 1976; Mohedano-Mariano et al., 2007; Keshavarzi et al., 2015) and indirectly from the main olfactory bulb *via* the cortical amygdala (Swanson and Petrovich, 1998). With strong connections to all nodes of the SBN, the MeA sits at the nexus of sensory and social behavioral processing. To understand the role of MeA in integrating information, it is important to appreciate the heterogeneity, distinct temporal dynamics of neural activity, and broad anatomical connections MeA circuits make with the rest of the brain.

The MeA is divided into four subregions with unique functional, cytoarchitectural and anatomical qualities (Swanson and Petrovich, 1998; Petrulis, 2020; Raam and Hong, 2021). Dorsal regions contain a higher abundance of inhibitory neurons, while ventral regions contain more excitatory neurons (Keshavarzi et al., 2014). Posterior regions, posterodorsal (MeApd) and posteroventral (MeApv), and the anterior MeA (MeAa) are distinguished by their response to reproductive and defensive stimuli, as well as their differential expression of transcription factors Lhx6, Lhx9, and Lhx5. For example, Lhx9 and Lhx5 expressing MeAa and the MeApv neurons preferentially respond to predation stimuli and form circuits with other defensive BNST and hypothalamic nodes, while Lhx6 neurons in the MeApd form reproductive circuits with the posterior region of the BNST and the hypothalamus (Choi et al., 2005). Molecular heterogeneity correlates with the behavioral outcomes of MeA circuits, and here we focus on the MeA circuits for reproduction and aggression.

The MeApd is known to produce many socially and reproductive relevant behaviors, including aggression (Newman, 1999). The MeApd is active during aggressive events (Kollack-Walker and Newman, 1995; Veening et al., 2005) and aggression in both sexes is suppressed by MeApd lesions (Kemble et al., 1984; Takahashi and Gladstone, 1988). Channelrhodopsin stimulation of GABAergic, and not glutamatergic, cell populations within the MeApd elevates aggressive behaviors (Hong et al., 2015). Similarly, aromatase-expressing MeApd neurons, aromatase being required for the conversion of testosterone to estradiol in the brain, regulate both intermale and maternal aggression (Unger et al., 2015). Currently, the overlap between GABAergic and aromatase-expressing MeApd neurons in the context of aggressive behavior is not clear and, therefore, it remains unknown whether these represent multiple aggression circuits or multiple nodes in a single aggression circuit in the MeA.

MeApd circuits have been linked to social behaviors beyond aggression. Activation of tachykinin-expressing GABAergic neurons in the MeApd promotes allogrooming of distressed social partners (Wu et al., 2021), while activation of glutamatergic neurons promotes asocial self-grooming (Hong et al., 2015). Sex discrimination, recognition and social memory have all been linked to the MeA and are dependent on oxytocin input to aromatase-expressing neurons (Ferguson et al., 2001; Yao et al., 2017). *In vivo* imaging in the form of micro endoscopes have now been used to longitudinally record MeA circuit function during specific social behaviors including social discrimination and avoidance/approach behavior, as well as through periods of plasticity including before and after sexual experience (Li et al., 2017; Miller et al., 2019). The findings from these studies show subsets of neurons functioning with distinct temporal dynamics, as well as sex differences in circuit function. Experiments that monitor the moment-to-moment activity of neurons during behavior, in line with previous studies, underscore the importance of neuronal heterogeneity as an organization principle that allows circuits to produce a large breadth of behavioral output.

**FIGURE 2:**
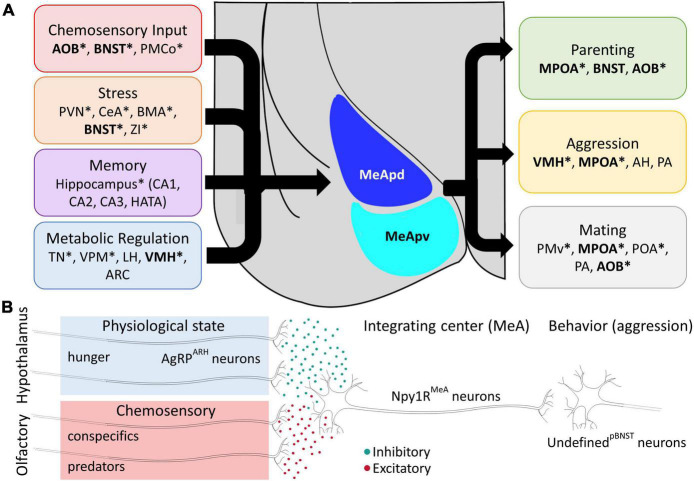
Structural and Functional Integration in Medial Amygdala Circuits. **(A)** Left: The MeA receives synaptic input from diverse circuits dedicated to stress, chemosensation, memory, and metabolic regulation. Right: Synaptic inputs are integrated in the MeA to direct innate aggressive, parental, and reproductive behaviors. Example connections represent the largest efferent and afferent connections but are not exhaustive. Bolded brain regions indicate regions with identified reciprocal connections (feedback loops) with the MeA. Connections indicated by an asterisk (*) are identified specifically for aromatase-expressing MeA neurons. Posteromedial cortical amygdala (PMCo), paraventricular nucleus of the hypothalamus (PVN), central amygdala (CeA), basomedial amygdala (BMA), zona incerta (ZI), hippocampal-amygdaloid transition area (HATA), ventral posteromedial nucleus (VPM), lateral hypothalamus (LH), arcuate nucleus of the hypothalamus (ARC), anterior hypothalamus (AH), posterior amygdala (PA) and premotor cortex (PMv) (Wu et al., 2009; Dwyer et al., 2022). **(B)** The MeA receives converging chemosensory and interoceptive input from the AOB and hypothalamus, respectively. During starvation conditions, AgRP neurons release inhibitory signals onto postsynaptic Npy1R neurons in the MeA. Excitatory chemosensory input from the AOB conveys information about predator and/or conspecific cues to the MeA. This dichotomy positions the MeA for scalable outcomes in a hierarchy of survival with respect to social behaviors. When a starving mouse is presented with a conspecific intruder, it prioritizes escape behaviors over territorial aggression, and this behavior is replicated by experimental activation of AgRP fibers in the MeA. AgRP neurons release a multitude of inhibitory signals. Fast acting GABA can act on a short order, while the inhibitory tone from the neuropeptides, NPY and AGRP signals can persist for days.

The MeA and, accordingly, the behaviors mediated by the MeA display clear sex differences in anatomy and function (Cooke and Woolley, 2005; Bergan et al., 2014). However, single cell scRNA-seq data suggests surprisingly few sex differences in the abundance of neurons in each major classes of MeA neurons. Instead, molecular variation in GABAergic neurons has been suggested to underlie the vast differences in behavioral outcomes between sexes (Wu et al., 2017; Chen et al., 2019). In aromatase-expressing neurons, there are clear sex differences, with males expressing roughly 50% more aromatase neurons in the MeApd (Morris et al., 2007; Wu et al., 2009; Yao et al., 2017). Given the role of aromatase neurons in driving aggression, a sexually dimorphic social behavior (Unger et al., 2015), this variation in local MeA circuit design creates a framework for understanding how behavior can adapt between types of individuals. In addition to genetic identity, anatomical connections represent another feature by which neurons can be classified with clear ramifications for understanding complex behavioral output. For example, MeA aromatase-expressing neurons receive conspecific pheromonal information *via* projections from the anterior AOB (Billing et al., 2020), reflecting a model of the MeA as the third step in a chemosensory pathway that produces social behaviors (Lee et al., 2014; Unger et al., 2015; Bayless and Shah, 2016; Ishii et al., 2017). However, aromatase-expressing neurons in the MeA receive inputs from dozens of subcortical brain regions, including regions associated with fear, memory, and metabolic regulation. Indeed, the “classic” chemosensory inputs from the AOB and inputs from the BNST each make up less than 2% of the total input to MeA-aromatase neurons (Dwyer et al., 2022), implying that this single subset of MeA neurons integrates sensory input with a broad array of internal physiological and learned information prior to signaling to behavioral control centers in the hypothalamus (Figure 2A).

The largest proportion of synaptic inputs to aromatase-expressing neurons in the MeA actually comes from other neurons *within* the MeA (Dwyer et al., 2022), suggesting that these neurons also integrate signals from neighboring local circuits containing different neuronal subtypes, which are in turn processing information from different compliments of brain regions. In many cases MeA neurons make reciprocal connections with other nodes in the SBN (e.g., BNST, MPOA, VMH, posterior amygdala and ventral premammillary nucleus) further highlighting the non-linear nature of the circuits mediating social behavior and demonstrating the complex orchestration of recursive feedback and broad integration at multiple levels from which complex social behaviors like parenting likely emerge.

## Circuit Connections at the Intersection of Divergent Behaviors

Physical integration of circuits such as we see in the MeA likely allows integration, not just between different social behaviors, but also between social and non-social behaviors. The relationships between seemingly divergent behaviors are reflected in the organization and connections between social and non-social circuits. Early on, neuroscience research on feeding demonstrated striking results using molecularly engineered receptors to gain control Agouti-related peptide (AgRP) neurons. AgRP neuronal soma are exclusively localized to the arcuate hypothalamus and play a vital role in mounting a hunger response. Toxin-induced ablation of AgRP neurons in adult mice led to lethal aphagia (Luquet et al., 2005). In 2011, the field applied opto- and chemogenetics to AgRP neurons. AgRP neuron stimulation was sufficient to evoke feeding behavior; well-fed animals consumed food as if they were starved (Aponte et al., 2011; Krashes et al., 2011). Direct stimulation of AgRP neurons evoked at least two aspects of starvation, the drive to consume food, and decreased energy expenditure. These experiments investigated a discrete behavioral outcome; the animals were removed from a social setting and food was available at little cost.

While discrete behaviors can be assessed in a controlled laboratory setting, the stimuli that drive behavior are rarely experienced in isolation in nature. To that end, the above experiments did offer some environmental context. AgRP stimulation was performed during the rest phase and therefore the act of feeding required arousal, calling into question the role of AgRP activity and by proxy hunger itself in a complex system of competing need states. Starving animals in the wild demonstrate both behavioral and physiological adaptations: fertility is gated, avoiding the costly energetic demands of reproduction (Iwasa et al., 2018); metabolism slows, requiring fewer calories to meet basal metabolic demands (Leibel, 1990); foraging strategies shift (Krebs, 1980); and food seeking activity is prioritized over rest/sleep (Alvarenga et al., 2005).

As a population, individual AgRP neurons have a near uniform response to fasting (Betley et al., 2015). AgRP neurons send projections, with few to no collaterals, to at least 14 downstream brain regions, fostering the idea that each projection played a “parallel and redundant” role in food seeking behavior (Broberger et al., 1998; Betley et al., 2013). However, because AgRP neurons were implicated in competing need state dynamics, it was also plausible to view them as a “distribution center,” receiving cues of low energy status and distributing this information to a variety of postsynaptic targets that contribute to the collective starvation-associated state change. The power of optogenetic fiber stimulation provided a means to test these hypotheses.

With respect to this review, it is important to consider that AgRP neurons send projections to multiple relays of the social behavioral network, and it is important to note that hunger can tune the expression of social behaviors. During starvation, food seeking efforts become dominant, to the extent that species of prey will migrate out of their protected territorial safe zone and seek food in areas that are more susceptible to predation (Krebs, 1980). Evidence of AgRP communication to the MeA was shown by projection mapping studies (Broberger et al., 1998; Padilla et al., 2016). Adding to this, inhibitory AgRP neurons co-express NPY and both NPY receptors along with melanocortin 4 receptors are expressed in the MeA, further highlighting the remarkable heterogeneity of MeA neurons (Figure 2B; Kishi et al., 2003; Liu et al., 2003; Padilla et al., 2016). It was proposed that the starved state may tune territorial aggression *via* communication from AgRP neurons to the MeA with the rationale that during starvation, the cost of territorial defense is outweighed by the animal’s primal drive to find and consume food. Stimulation of AgRP fibers in the MeA was sufficient to reduce territorial aggression of resident males toward subordinate conspecific male intruders (Padilla et al., 2016). In alignment with a shift in priorities, stimulation of AgRP fibers in the MeA produced more escape behaviors in the presence of an intruder indicating a reduced motivation for antagonistic social interactions (Padilla et al., 2016). In this experimental paradigm, the MeA receives converging input from at least two stimuli and demonstrates the power of a dominant need state in decision making. Indeed, social circuit function is fundamentally altered by AgRP neuron signaling through a collective group of physiological changes that are attributed to starved state adaptations in wild animals (Dietrich et al., 2015; Burnett et al., 2016, 2019; Jikomes et al., 2016; Padilla et al., 2017; Alhadeff et al., 2018; Goldstein et al., 2018; Xia et al., 2021).

An appreciation for the importance of behavioral integration has similarly grown out of research on the circuits that control pain sensitivity. The initial discovery that descending projections from the brainstem were capable of endogenously modulating pain set the stage for decades of fruitful research into the role of raphe magnus neurons in gating an animal’s sensitivity to painful stimuli (Basbaum et al., 1978; Basbaum and Fields, 1978). Raphe neurons project to the dorsal horn of the spinal cord and the activities of raphe neurons modulate sensitivity to painful stimuli through two opposing groups of neurons. ON cell activation upregulates sensitivity to pain while OFF cell activation decreases sensitivity to pain (Mason and Fields, 1989; Skinner et al., 1997) — collectively enabling bidirectional control of pain sensitivity. Subsequent research into raphe magnus function investigated the behavioral conditions during which ON and OFF cells are active and found that the current behavioral state dramatically influenced activity in the raphe magnus (Leung and Mason, 1999). Behavioral states including sleep, feeding, and sex correlate with both reduced pain sensitivity and activation of raphe magnus OFF cells (Mason, 2001). Thus, while raphe magnus neurons are essential for modulating sensitivity to pain, descending pain modulation may be best understood in service of defending critically important behavioral states such as sleep, sex, and feeding from interruption. Indeed, raphe magnus neurons have access to information about sex, feeding, and sleep and, collectively, these behavioral states provide the context for how pain sensitivity is regulated on a moment-to-moment basis.

## Neuromodulation of Behavior Circuits

**FIGURE 3:**
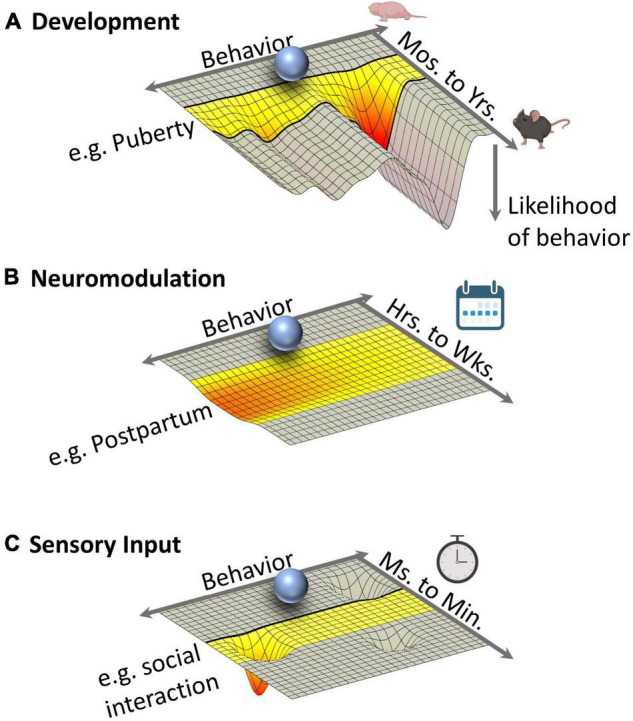
Multiple Influences on the Energy Landscape of Social Circuit Function. Each energy landscape represents the probability of behavioral outcomes over three different timescales. Highlighted periods of time represent epochs during which circuit functions change, transiently or permanently, because of developmental, neuromodulatory, or sensory and interoceptive events. The depth of a given “energy well” is proportional to the likelihood of a specific behavior with deep troughs representing highly probably behaviors. Ultimately, the output of a social circuit represents the integration of these energy constraints. **(A)** Development. Puberty represents a developmental transition point during which the energy landscape of social circuits is dramatically reorganized to favor adult behaviors. The resulting energy landscape is, in large part, persistent through life. **(B)** Neuromodulation. Changes in neuromodulatory tone (e.g., steroid hormones or neuropeptides), such as those that occur during the postpartum period, persist for hours to weeks during which a subtle change to the energy landscape alters the likelihood of multiple behaviors. **(C)** Sensory input. Rapid fluctuations (milliseconds to minutes) in the energy landscape of social circuits are driven by dynamic and transient sensory and interoceptive input including the transduction of sensory cues during social interactions. Time symbols created with BioRender.com.

Thus far, we have discussed how neurons connect locally and brain-wide to form social circuits. But social circuits also vary between individuals and over a range of timescales. For example, the sensory stimuli that drive strong responses in MeA neurons differ between males and females and also change as an animal ages (Morris et al., 2007; Bergan et al., 2014; Yao et al., 2017). Indeed, many social circuits undergo developmental organization and activation phases during which the probabilistic landscape of potential behaviors is remodeled (Figure 3A). And among the physical connections between neurons are modulatory systems that can alter the sensitivity of individual neurons within the circuit, making them more or less prone to firing, and thereby influencing which behaviors a circuit produces.

Neuromodulatory effects are caused by the action of a wide range of signaling molecules, including molecules like serotonin and dopamine that can also act as neurotransmitters, neuropeptides like vasopressin and oxytocin, and steroid hormones like testosterone and estradiol (Remage-Healey, 2014). Neuromodulators may arrive from peripheral sources *via* the circulatory system or be secreted locally by neurons, reaching their targets either directly by synaptic transmission or indirectly by volume transmission (Nadim and Bucher, 2014). Some bind to receptors in the cell membrane, triggering second-messenger systems that change neuron activity on timescales on the order of minutes to hours (Taxier et al., 2020), others, particularly steroid hormones, may produce longer-term effects by modifying gene expression (Kelly et al., 2013; Campbell and Herbison, 2014; Nadim and Bucher, 2014). Importantly, neuromodulatory signaling, including both neuropeptides and steroid hormones, is rife in the SBN and likely affects nearly all circuits devoted to social behavior.

Because neuromodulators change how neurons behave within a circuit but have little effect on the physical structure of that circuit, they can induce transient dynamic changes within circuits in response to specific changes in internal and external environments on timescales longer (hours to weeks) than traditional neurotransmission (Figure 3B; Marder, 2012; Remage-Healey, 2014). This flexibility lets animals shift temporarily from one behavioral state to another: for example, changes to local estradiol levels in brain tissue rapidly affects male sexual motivation in quail (Cornil et al., 2018); in mice, higher progesterone levels during diestrus preferentially dampen the activity of VNO neurons that detect male pheromones, turning off female courtship and copulatory behaviors until progesterone levels drop during estrus (Dey et al., 2015). Moreover, because the action of each neuromodulatory molecule depends on receptor binding, its effects are targeted to only the subset of neurons in a circuit that express the specific receptors it binds to.

Neuromodulation in a circuit can be complex. The strength of any specific neuromodulator’s effect on its target neurons can change in concert with local concentrations of that molecule, as demonstrated by the effects of aromatase inhibitors on estradiol-mediated sexual behaviors in quail (Cornil et al., 2006), goldfish (Lord et al., 2009), and mice (Taziaux et al., 2007), and on aggressive behaviors in mice and birds (Heimovics et al., 2015). Upregulation or downregulation of a neuromodulatory receptor can have similar effects (Kenakin, 2004). Complicating things even more, individual neurons can express more than one type of neuromodulatory receptor (Shughrue et al., 1998; Ochiai et al., 2004; Nadim and Bucher, 2014), and the neurons that populate a circuit can be heterogeneous, with each expressing receptors for a different suite of neuromodulators. Furthermore, neuron activity may also be modified by signals from adjacent glia, which may also be subject to neuromodulatory molecules (Wahis et al., 2021). This means that a circuit can be simultaneously subject to multiple types of modulation, whose actions may produce opposing effects (Ogawa et al., 1997, 1999) or potentially complement one another.

Experimentally observing the effects of neuromodulation on circuits is understandably challenging. But new *in vivo* technological advances in electrophysiology paired with genetic targeting and activity markers open the possibility of observing circuit and network dynamics in real time. One such example includes micro endoscopes and fiber photometry paired with calcium imaging (Aharoni and Hoogland, 2019). With genetically targeted activity markers such as GCaMP, RCaMP, and the like it is possible to observe sensory responses, circuit activity, and network dynamics in an area of interest (Figure 3C). These techniques will allow researchers to observe many neurons over multiple behavioral events, for weeks up to months and, therefore, make it possible to gauge the effects of neuromodulation on circuit function over time.

## Conclusion

Tremendous progress has been made toward associating the activities of genetically defined populations of neurons in distinct brain regions with specific social behaviors. Emerging from this collective work is a complex image of brain circuits in which the structural and molecular heterogeneity of neurons is not an experimental problem to overcome, but rather, a feature that provides precise access to unlock the underlying logic of individual social behaviors. Genetic approaches often leverage neuroanatomical, behavioral, and functional approaches for a comprehensive and multifaceted description of brain circuits and provide unprecedented predictive power over the behavioral consequences of neural activity. At the same time, it is important to appreciate the experimental conditions that produce our understanding of these circuit functions. For example, the same genetically defined neuron population can drive or suppress aggression when activated with different temporal dynamics (Lee et al., 2014; Baleisyte et al., 2021), demonstrating that the dynamics of activation patterns can profoundly shape behavioral outcomes. As endogenous patterns of neural activity are revealed by techniques such as single cell *in vivo* fluorescent imaging, progress on optogenetics suggests single cell functional manipulations may eventually become feasible (Papagiakoumou et al., 2009, 2020). To this point, however, the temporal profiles used to experimentally manipulate social circuits typically have not replicated the endogenous activity patterns. Thus, while the causal relationships between the activation of a specific neuron population and the resulting behavior are undeniable, testing the impact of endogenously realistic patterns of activity on behavioral output remains challenging.

Genetic approaches allow reproducible access to a precisely defined population of neurons (Choi et al., 2005; Wu et al., 2014), and experimental control over the activity of these neurons (Boyden et al., 2005; Armbruster et al., 2007). However, social circuits display profound integration on multiple spatial and temporal scales. For example, significant progress has been made toward identifying the molecular and structural organization of circuits responsible for individual social behaviors, and toward identifying the neuromodulatory nodes that influence these behaviors. However, in most cases it remains unclear how neuromodulatory input remaps the probabilistic relationships between sensory input and the behaviors they promote. Do the same neurons mediate social behaviors regardless of interoceptive states? Is the output or electrophysiological activity of a behavioral circuit suppressed or enhanced by a particular neuromodulator? We are just now starting to understand how fluctuations in neuromodulatory state alter circuit dynamics, and the resulting behaviors, on a moment-to-moment basis (Falkner et al., 2014; Tang et al., 2020). Moreover, the effects of neuromodulation depend on how social circuits are organized during development, and therefore the effects of neuromodulation often differ between individuals based on factors such as age, sex or experience (McCarthy et al., 2012; de Vries et al., 2014). Understanding individual differences in the organization of social behaviors is a particularly important goal.

The profound integration occurring at social circuits in the brain (e.g., Dwyer et al., 2022), trumpets the interdependent nature of these critically important behaviors. Thus, the probability of a specific social behavior depends on the identity of both social partners and interoceptive states, such as hunger, that require a shift in the landscape of potential social behaviors (Padilla et al., 2016). As such, a behavioral hierarchy emerges in which hunger suppresses social interaction and sensitivity to pain, while social interactions are suppressed by hunger but suppress an animal’s sensitivity to pain. The relationships between different behavioral states are reflected in the organization of the underlying circuits and, in some cases, the organization of social circuits can inform our understanding of behavior (Mason, 2001; Padilla et al., 2016). While we have discussed this primarily in the context of social behavior, it is our belief that profound integration is a feature of many brain circuits and progress toward understanding the native function beyond social circuits will often require establishing the relationships between different behaviors.

In many cases, the tools required to investigate the connections *between distinct behavioral* circuits are already available. Viral anatomical tracing methods provide ways to identify the brain-wide afferent (Wickersham et al., 2007; Menegas et al., 2015; Dwyer et al., 2022), and efferent (Feinberg et al., 2008; Wu et al., 2009; Ishii et al., 2017) synaptic partners to a genetically and anatomically defined population. Intersectional genetic approaches allow simultaneous investigation of multiple genetically defined cell populations (Austin et al., 2004; Knobloch et al., 2012; Ishii et al., 2017; Carcea et al., 2021). At the same time, the ability to monitor the activities of many, well-defined, neurons continues to accelerate (Lee et al., 2014; Li et al., 2017; Menegas et al., 2018). If recent progress characterizing the basis for individual social behaviors at the level of genetically defined neural circuits serves as a valid benchmark, the coming years will provide remarkable insights into how distinct behavioral circuits integrate information from disparate circuits.

## Author Contributions

SD, DK, SP, and JB conceived of the topic, wrote the manuscript, and edited the manuscript together. All authors contributed to the article and approved the submitted version.

## Conflict of Interest

The authors declare that the research was conducted in the absence of any commercial or financial relationships that could be construed as a potential conflict of interest.

## Publisher’s Note

All claims expressed in this article are solely those of the authors and do not necessarily represent those of their affiliated organizations, or those of the publisher, the editors and the reviewers. Any product that may be evaluated in this article, or claim that may be made by its manufacturer, is not guaranteed or endorsed by the publisher.
